# Trichlorido(tetra­hydro­furan){(1,2,3,3a,7a-η)-1-[2-(1-trimethyl­silyl-1*H*-imidazol-2-yl-κ*N* ^3^)-1-methyl­prop­yl]inden­yl}zirconium(IV)

**DOI:** 10.1107/S1600536811012037

**Published:** 2011-04-07

**Authors:** Shengzhou Guan, Wanli Nie, Maxim V. Borzov

**Affiliations:** a Key Laboratory of Synthetic and Natural Chemistry of the Ministry of Education, College of Chemistry and Material Science, the North-West University of Xi’an, Taibai Bei Avenue 229, Xi’an 710069, Shaanxi Province, People’s Republic of China

## Abstract

The title compound, [ZrCl_3_(C_19_H_25_N_2_Si)(C_4_H_8_O)], was prepared from bis­(*N*,*N*-dimethyl­amido-κ*N*)(2-{2-[(1,2,3,3a,7a-η)-inden­yl]-2-methyl­prop­yl}-1*H*-imidazolido-κ*N* ^1^)zirconium(IV) [(C_16_H_16_N_2_)Zr(NMe_2_)] by reaction with excess Me_3_SiCl in tetra­hydro­furan (THF) at elevated temperature. The crystal studied contained a minor non-merohedral twin contaminant [6.3 (4)%] which was taken into account during the refinement. The coordination polyhedron of the Zr^IV^ atom is a distorted octa­hedron [assuming that the five-membered ring of the indenyl group (Cp) occupies one coordination site], with the Cp group and a THF O atom at the apical positions and the three Cl and ligating N atoms at the equatorial positions. The Zr, Si and the methyl­ene C atoms deviate noticeably from the imidazole ring plane [by −0.197 (5), −0.207 (5) and 0.119 (6) Å, respectively]. The THF ligand adopts an envelope conformation.

## Related literature

For general practical utility of geometry-constrained complexes, including those derived from group 4 transition metals, see: Erker (2006 ▶); Braunschweig & Breitling (2006 ▶). For the geometric parameters of similar Zr^IV^ complexes, see: Nifant’ev *et al.* (1998 ▶); Paolucci *et al.* (2003 ▶); Krut’ko *et al.* (2004 ▶, 2007 ▶); Enders *et al.* (1996 ▶); Nie *et al.* (2008 ▶). For Ti^IV^ analogues of the title compound, see: Ge *et al.* (2010 ▶) and references cited therein. For procedures used in the preparation, see: Curtis & Brown (1980 ▶); Chisholm *et al.* (1988 ▶); Diamond *et al.* (1996 ▶); Weizmann *et al.* (1950 ▶); Armarego & Perrin (1997 ▶). For a description of the Cambridge Structural Database, see: Allen (2002 ▶). 
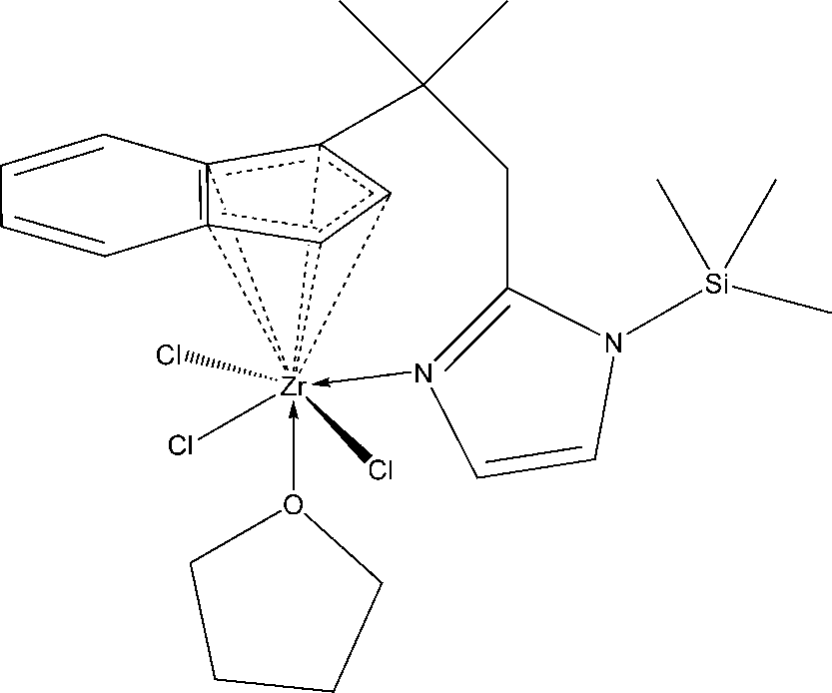


## Experimental

### 

#### Crystal data

[ZrCl_3_(C_19_H_25_N_2_Si)(C_4_H_8_O)]
*M* *_r_* = 579.17
Triclinic, 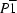

*a* = 10.6274 (8) Å
*b* = 10.9496 (7) Å
*c* = 13.1397 (9) Å
α = 102.720 (1)°
β = 101.416 (1)°
γ = 110.456 (1)°
*V* = 1332.73 (16) Å^3^
*Z* = 2
Mo *K*α radiation
μ = 0.78 mm^−1^
*T* = 296 K
0.35 × 0.24 × 0.14 mm

#### Data collection

Bruker SMART APEXII diffractometer
Absorption correction: multi-scan (*TWINABS*; Sheldrick, 2006 ▶) *T*_min_ = 0.773, *T*_max_ = 0.899
4871 measured reflections
4871 independent reflections
3757 reflections with *I* > 2σ(*I*)

#### Refinement

*R*[*F* ^2^ > 2σ(*F* ^2^)] = 0.037
*wR*(*F* ^2^) = 0.093
*S* = 1.00
4871 reflections
286 parameters
H-atom parameters constrained
Δρ_max_ = 0.42 e Å^−3^
Δρ_min_ = −0.52 e Å^−3^

### 

Data collection: *APEX2* (Bruker, 2007 ▶); cell refinement: *SAINT* (Bruker, 2007 ▶); data reduction: *SAINT*; program(s) used to solve structure: *SHELXS97* (Sheldrick, 2008 ▶); program(s) used to refine structure: *SHELXL97* (Sheldrick, 2008 ▶); molecular graphics: *SHELXTL* (Sheldrick, 2008 ▶) and *OLEX2* (Dolomanov *et al.*, 2009 ▶); software used to prepare material for publication: *SHELXTL* and *OLEX2*.

## Supplementary Material

Crystal structure: contains datablocks I, global. DOI: 10.1107/S1600536811012037/dn2668sup1.cif

Structure factors: contains datablocks I. DOI: 10.1107/S1600536811012037/dn2668Isup2.hkl

Additional supplementary materials:  crystallographic information; 3D view; checkCIF report

## Figures and Tables

**Fig. 1.**
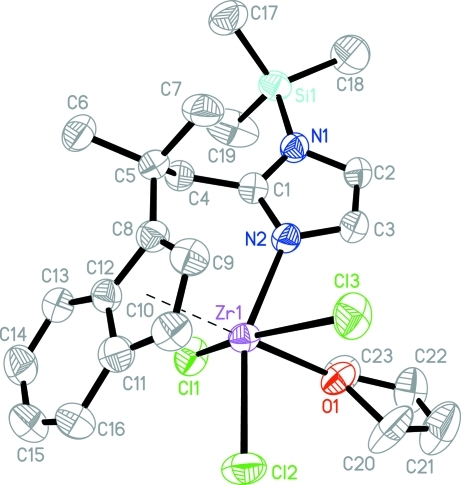
Unsymmetrical unit of I with labeling. Thermal ellipsoids are shown at the 50% level of probability. All H-atoms are omitted for clarity. The Zr1 to Cp-centroid bond is depicted as a dashed line.

## supplementary crystallographic information

### Comment

The title compound, C_23_H_33_Cl_3_N_2_OSiZr, I, relates to the family of so-called geometry constrained complexes what find their application for the catalytic ethylene and α-olefin polymerization (including the stereospecific one; for general information, see reviews: Erker, 2006; Braunschweig & Breitling, 2006). It has been prepared from bis(*N*,*N*-dimethylamido-κ*N*)(2-{2-[(1,2,3,3a,7a-η)-indenyl]-2-methylpropyl}-1*H*-imidazolido-κ*N*^1^)zirconium(IV), (C_16_H_16_N_2_)Zr(NMe_2_)_2_, II, by a reaction with excess of Me_3_SiCl in THF at elevated temperature (see the Experimental section for further details). The sample crystal of I contained a minor non-mehrohedral twin contaminant [6.3 (4)%] what was taken into account during the refinement (see the Refinement details section).

The coordination polyhedron of the Zr-atom in I is a distorted octahedron [assuming that the five-member ring of the indenyl group (Cp) occupies one coordination site], with the Cp-group and O-atom of the tetrahydrofuran (THF) molecule at the apical positions and the three Cl- and ligating N-atoms at the equatorial ones (Fig. 1). The Zr-, Si- and the methylene group C-atoms noticeably deviate from the imidazole ring plane [by -0.197 (5), -0.207 (5) and 0.119 (6) Å, respectively]. Indenyl group is planar within 0.06 Å. The THF ligand adopts an envelop conformation.

Analysis of the Cambridge Structural database (CSD; Version 5.27, release May 2009; Allen, 2002) reveals only 7 structurally characterized Zr^IV^ complexes of the similar to I (η^5^-Cp-link-N*R*_n_-κ*N*)ZrCl_3_ type (8 independent fragments). Among them, there are two dinuclear structures where two Zr-atoms are linked with two bridging µ-Cl-atoms (Enders *et al.*, 1996 and Nie *et al.*, 2008; in both cases Zr-atoms exhibit *CN* 6), two monomeric complexes with a pentacoordinated Zr centre (Nifant'ev *et al.*, 1998 and Krut'ko *et al.*, 2004), and, finally, three monomeric complexes with a hexacoordinated Zr centre (Paolucci *et al.*, 2003; Krut'ko *et al.*, 2004; Krut'ko *et al.*, 2007). Of interest and despite of the different nature of the "sixth" *n*-donor ligand opposing the Cp-group [a tetrahydrothiophene molecule (Krut'ko *et al.*, 2004), a pyridine molecule (Krut'ko *et al.*, 2007), or a pendant OH-group (Paolucci *et al.*, 2003)], the structural motif of the latter three complexes is very similar to that of I. As for the nature of the Cp-type ligand, only one case among all the mentioned above corresponds to an indenyl group (Nifant'ev *et al.*, 1998).

As it was observed earlier for Ti-analog of I (Ge *et al.*, 2010), in a THF solution I co-exists with a mixture of Me_3_SiCl and drichloro(2-{2-[(1,2,3,3a,7a-η)-indenyl]-2-methylpropyl}-1*H*-imidazolido-κ*N*^1^)zirconium(IV), (C_16_H_16_N_2_)ZrCl_2_, III, (for spectral proof, see Experimental).

### Experimental

All operations were performed under argon atmosphere in conventional glassware or in all-sealed evacuated glass vessels with application of the high-vacuum line (the residual pressure of non-condensable gases within 1.5–1.0.10 ^-3^Torr; 1 Torr = 133 Pa). 1-(1-Methylethylidene)-1*H*-indene was prepared as described by Weizmann *et al.*, 1950. 1-Diethoxymethyl-2-methyl-1*H*-imidazole and its lithiated derivative were prepared by a close analogy to what described by Curtis & Brown, 1980. Bis[µ-(*N*-methylmethanamido-κ^2^*N*)]hexakis(*N*-methylmethanamido-κ*N*)dizirconium, [Zr(NMe_2_)_3_]_2_(µ-NMe_2_)_2_, was prepared as described by Chisholm *et al.*, 1988 and Diamond *et al.*, 1996. A l l other chemicals were commercially available and purified by conventional methods (Armarego & Perrin, 1997). Solvents were purified by distillation over sodium benzophenoneketyl (diethyl ether, THF), Na—K alloy (toluene), and CaH_2_ (chloroform and dichloromethane). Deuterated solvents were dried similarly. — NMR spectra were recorded on a Varian INOVA-400 instrument. For ^1^H and ^13^C spectra, the solvent [δ_H_ = 1.73 and δ_C_ = 25.3 (THF-*d_8_*)] or TMS (δ_H_ = 0.00 and δ_C_ = 0.0) (CDCl_3_) resonances were used as internal reference standards. — Chromato-mass spectra were measured on Agilent 6890 Series GC system equipped with HP 5973 mass-selective detector. — The elemental analyses were performed on the Vario ELIII CHNOS automated analyzer.

1-Diethoxymethyl-2-methyl-1*H*-imidazole, IV: 2-Methyl-1*H*-imidazole (24.6 g, 0.3 mol), triethyl orthoformate (178.2 g, 1.2 mol), and *p*-toluenesulfonic acid (0.9 g) were heated under Ar at 403 K in a distillation flask equipped with a short (10 cm) Vigreux column until no more ethanol was distillable from the reaction mixture. The excess of triethyl orthoformate was removed by distillation under reduced pressure, 1 g of solid Na_2_CO_3_ was added, and the residue was fractionally distilled to give 41.7 g of IV (75%) as a colorless flexible oil. B. p. 333–336 K (82–85 – Pa). Yield 75%. – ^1^H NMR (300.5 K, CDCl_3_): δ = 1.24 (t, *X*_3_ part of AB*X*_3_ spin system, ^3^*J*_AX_ = ^3^*J*_BX_ = 7.2 Hz, 6H, CH_3_ in Et-group), 2.45 (s, 3H, 2-CH_3_), 3.55, 3.59 (m, AB part of AB*X*_3_ spin system, ^3^*J*_AX_ = ^3^*J*_BX_ = 7.2 Hz, ^2^*J*_AB_ = 9.28 Hz, 4H, CH_2_), 5.98 (s, 1H, CH(OEt)_2_), 6.90, 7.10 (both s, 1H + 1H, CH=CH). – ^13^C{^1^H} NMR (300.5 K, CDCl_3_): δ = 13.49 (2-CH_3_–C_imid_), 14.40 (H_3_*C*–CH_2_O), 61.06 (H_3_C–*C*H_2_O), 100.80 (CH(OEt)_2_), 116.29, 126.48 (CH=CH), 143.54 (N=C—N). EI MS (70 eV) *m/z* (%): 184 (5.2) [*M*]^+.^, 139 (32.9) [*M* – OEt^.^]^+^, 111 (28.0) [*M* – OEt^.^ –C_2_H_4_]^+^, 103 (100.0) [HC(OEt)_2_]^+^, 83 (32.9) [2-CH_3_–C_3_N_2_H_4_]^+^, 82 (23.2) [2-CH_3_–C_3_N_2_H_3_]^+^, 81 (47.9) [2-CH_3_–C_3_N_2_H_2_]^+^, 75 (58.1) [HC(OEt)_2_ – C_2_H_4_]^+^, 54(19.5) [C_3_H_4_N]^+^. — Elemental analysis for III failed due to its extreme sensitivity to air moisture.

(1-Diethoxymethyl-1*H*-imidazol-2-yl)methyllithium, V: To a solution of protected imidazole IV (10.20 g, 50.0 mmol) in THF (150 ml), *n*-BuLi (32 ml of 1.87 *M* solution in hexane, 60.0 mmol) was added *via* a syringe at -40°C under vigorous stirring. After the addition complete, the red–brown solution was kept at -40°C for additional 15 min prior to use.

2-[2-(1*H*-inden-3-yl)-2-methylpropyl]-1*H*-imidazole, VI: A solution of 1-(1-methylethylidene)-1*H*-indene (9.20 g, 60.0 mmol) in THF (60 ml) was added to the solution of V in THF (see above) during 30 min at -40°C. After 5 min at -40°C the cooling bath was removed, the mixture was allowed to warm gradually up to room temperature and left to stay overnight. The mixture was diluted with diethyl ether (100 ml), cooled in an ice bath, and extracted with 0.5 N HC1 (4 portions each 50 ml). The combined acid extracts (pH 2) were neutralized with solid NaHCO_3_, extracted with CH_2_C1_2_ (3 ×100 mL) and dried with MgSO_4_. Removal of solvent under reduced pressure yielded VI (9.67 g, 68.9%) as light-yellow crystalline powder. — ^1^H NMR (298 K,CDCl_3_): δ = 1.41 [s, 6 H, C(CH_3_)_2_], 3.26 (s, 2 H, CH_2_ in the bridge), 3.32 (d, 2 H, ^3^*J* = 1.8 Hz, CH_2_ in indene), 6.20 (t, 1 H, ^3^*J* = 1.8 Hz, H2 in indene), 6.81 (broadened s, 2 H, CH in imidazole), 7.24, 7.32 (both m, both 1 H, H5 and H6 in indene), 7.52, 7.70 (both m, both 1 H, H4 and H7 in indene). — ^13^C{^1^H} NMR (298 K, CDCl_3_) δ = 27.40 [C(*C*H_3_)_2_], 37.10, 37.32 (CH_2_), 39.41 [*C*(CH_3_)_2_], 121.39 (broad, CH in imidazole), 121.79 (broadened, C2 in indene), 124.38, 124.48, 126.01, 128.80 (C4–7 in indene), 143.04, 145.66, 145.97 (quaternary C in indene), 151.01 (quaternary C in imidazole). — EI MS (70 eV) *m/z* (%): 238 (23) [*M*]^+.^, 223 (30) [M–CH_3_^.^]^+^, 157 (15) [C_12_H_13_]^+^, 156 (13) [C_12_H_12_]^+.^, 142 (42) [C_11_H_10_]^+.^, 141 (31) [C_11_H_9_]^+^, 115 (25) [C_9_H_7_]^+^, 82 (100) [C_4_H_6_N_2_]^+.^.

Bis(*N*,*N*-dimethylamido-κ*N*)(2-{2-[(1,2,3,3a,7a-η)-indenyl]-2-methylpropyl}-1*H*-imidazolido-κ*N*^1^)zirconium(IV), (C_16_H_16_N_2_)Zr(NMe_2_)_2_, II: [Zr(NMe_2_)_3_]_2_(µ-NMe_2_)_2_ (0.73 g, 1.39 mmol) and V (0.66 g, 2.77 mmol) in toluene (10 ml) were mixed and heated in an oil bath (353 K) for 18 h. On cooling, the reaction mixture was concentrated, the light-brown precipitate was filtered off from dark-red mother liquor, washed on a filter with small portions of cold toluene till colorless washings and dried on the high-vacuum line what gave II as light-brown crystalline material (1.09 g, 94.8%). — ^1^H NMR (THF-*d*_8_, 20°C): δ = 1.37 [s, 12 H, N(CH_3_)_2_], 2.30, 2.31 [both s, both 3 H, C(CH_3_)_2_], 3.06, 3.26 (both broadened s, both 1 H, CH_2_), 6.25 (m, 2 H, H2 and H3 in indene), 6.79, 6.81 (both m, both 1 H, CH in imidazole), 7.12, 7.23 (both m, both 1 H, H5 and H6 in indene), 7.42, 7.70 (both m, both 1 H, H4 and H7 in indene). Measurement of ^13^C{^1^H} for II was problematic due to its poor solubility even in THF.

Trichloro(tetrahydrofuran){1-[2-(1-trimethylsilyl-1*H*-imidazol-2-yl-κ*N*^3^)-1-methylpropyl]-(1,2,3,3a,7a-η)-indenyl}zirconium(IV), C_23_H_33_Cl_3_N_2_OSiZr, I: To a solution of II (1.00 g, 2.41 mmol) in THF (20 ml), excess of Me_3_SiCl (1.0 ml, 7.86 mmol) was added and the reaction mixture was then heated at 353 K during 8 h. Concentration of the mixture at ambient temperature gave yellow crystalline material. The product I was collected, washed with cold toluene till colourless washings and then dried on the high-vacuum line. Yield 0.63 g (45%). — ^1^H NMR (THF-*d*_8_, 298 K): δ = 0.41 [s, 9 H, Si(C*H*_3_)_3_ in chlorotrimethylsilane], 0.62 [s, 9 H, Si(C*H*_3_)_3_ in I], 0.94, 1.61 [both s, both 3 H, C(C*H*_3_)_2_ in III], 0.96, 1.65 [both s, both 3 H, C(C*H*_3_)_2_ in I], 3.02, 3.65 (both d, both 1 H, CH_2_ in III), 3.18, 3.76 (both d, both 1 H, CH_2_ in I), 6.52, 6.57, 6.99 (all m, H2 and H3 in indenyl in I and III), 7.05, 7.22 (both m, H5 and H6 in indenyl in I and III), 7.38, 7.47 (both m, CH in imidazole in I and III), 7.62, 7.79 (both m, H4 and H7 in indenyl in I and III).

Sample crystal of I suitable for X-ray diffraction analysis was grown up from hot THF and mounted inside a Lindemann glass capillary (diameter 0.5 mm; N_2_-filled glove-box).

### Refinement

The sample crystal of I contained a minor non-merohedral twin contaminant [dominant to minor component transform twin law (matrix row by row): 0.94452 - 0.10595 - 0.05098 0.07590 1.01441 - 0.03026 0.08528 0.09700 1.02798]. The absorption correction was processed with *TWINABS* (Sheldrick, 1996). The contribution of the minor component was estimated to be 5.55%. The structure was then solved with the detwinned HKLF 4 data file and finally refined with the HKLF 5 format data file with only single and composite reflections relating to the main component included and merged according to the point group -1 [the BASF parameter converges to 0.063 (4)]. Non-H atoms were refined anisotropically. H atoms were treated as riding atoms with distances C—H = 0.96 (CH_3_), 0.97 (CH_2_), and 0.93 Å (C_Ar_H) and *U*_iso_(H) = 1.5 *U*_eq_(C), 1.2 *U*_eq_(C), and 1.2 *U*_eq_(C), respectively.

### Crystal data

**Table:**

[ZrCl_3_(C_19_H_25_N_2_Si)(C_4_H_8_O)]	*Z* = 2
*M**_r_* = 579.17	*F*(000) = 596
Triclinic, *P*1	*D*_x_ = 1.443 Mg m^−^^3^
Hall symbol: -P 1	Mo *K*α radiation, λ = 0.71073 Å
*a* = 10.6274 (8) Å	Cell parameters from 7558 reflections
*b* = 10.9496 (7) Å	θ = 2.2–30.5°
*c* = 13.1397 (9) Å	µ = 0.78 mm^−^^1^
α = 102.720 (1)°	*T* = 296 K
β = 101.416 (1)°	Block, yellow
γ = 110.456 (1)°	0.35 × 0.24 × 0.14 mm
*V* = 1332.73 (16) Å^3^	

### Data collection

**Table:**

Bruker SMART APEXII diffractometer	4871 independent reflections
Radiation source: fine-focus sealed tube	3757 reflections with *I* > 2σ(*I*)
graphite	*R*_int_ = 0.000
Detector resolution: 8.333 pixels mm^-1^	θ_max_ = 25.5°, θ_min_ = 1.7°
phi and ω scans	*h* = −12→12
Absorption correction: multi-scan (TWINABS; Sheldrick, 1996)	*k* = −13→12
*T*_min_ = 0.773, *T*_max_ = 0.899	*l* = 0→15
4871 measured reflections	

### Refinement

**Table:**

Refinement on *F*^2^	Primary atom site location: structure-invariant direct methods
Least-squares matrix: full	Secondary atom site location: difference Fourier map
*R*[*F*^2^ > 2σ(*F*^2^)] = 0.037	Hydrogen site location: inferred from neighbouring sites
*wR*(*F*^2^) = 0.093	H-atom parameters constrained
*S* = 1.00	*w* = 1/[σ^2^(*F*_o_^2^) + (0.0479*P*)^2^] where *P* = (*F*_o_^2^ + 2*F*_c_^2^)/3
4871 reflections	(Δ/σ)_max_ = 0.001
286 parameters	Δρ_max_ = 0.42 e Å^−^^3^
0 restraints	Δρ_min_ = −0.52 e Å^−^^3^

### Special details

**Table:**

Experimental. 28 0.09700 1.02798].
Geometry. All e.s.d.'s (except the e.s.d. in the dihedral angle between two l.s. planes) are estimated using the full covariance matrix. The cell e.s.d.'s are taken into account individually in the estimation of e.s.d.'s in distances, angles and torsion angles; correlations between e.s.d.'s in cell parameters are only used when they are defined by crystal symmetry. An approximate (isotropic) treatment of cell e.s.d.'s is used for estimating e.s.d.'s involving l.s. planes.
Refinement. Refinement of *F*^2^ against ALL reflections. The weighted *R*-factor *wR* and goodness of fit *S* are based on *F*^2^, conventional *R*-factors *R* are based on *F*, with *F* set to zero for negative *F*^2^. The threshold expression of *F*^2^ > σ(*F*^2^) is used only for calculating *R*-factors(gt) *etc*. and is not relevant to the choice of reflections for refinement. *R*-factors based on *F*^2^ are statistically about twice as large as those based on *F*, and *R*- factors based on ALL data will be even larger.

### Fractional atomic coordinates and isotropic or equivalent isotropic displacement parameters (Å^2^)

**Table:**

	*x*	*y*	*z*	*U*_iso_*/*U*_eq_	
Zr1	0.61824 (3)	0.79166 (3)	0.25770 (2)	0.03334 (11)	
Cl1	0.58498 (10)	0.81107 (9)	0.07182 (6)	0.0502 (2)	
Cl2	0.41444 (10)	0.56691 (9)	0.19730 (8)	0.0583 (3)	
Cl3	0.72883 (11)	0.73742 (10)	0.41743 (7)	0.0576 (3)	
Si1	1.16456 (10)	1.26377 (10)	0.27386 (8)	0.0435 (2)	
O1	0.7140 (2)	0.6414 (2)	0.17924 (17)	0.0433 (6)	
N1	1.0468 (3)	1.1115 (2)	0.2942 (2)	0.0372 (6)	
N2	0.8520 (3)	0.9395 (2)	0.2879 (2)	0.0374 (6)	
C1	0.9063 (3)	1.0650 (3)	0.2797 (2)	0.0359 (7)	
C2	1.0824 (4)	1.0052 (3)	0.3115 (3)	0.0446 (8)	
H2	1.1715	1.0049	0.3227	0.054*	
C3	0.9655 (4)	0.9038 (3)	0.3092 (3)	0.0441 (8)	
H3	0.9608	0.8215	0.3202	0.053*	
C4	0.8250 (3)	1.1502 (3)	0.2676 (3)	0.0394 (8)	
H4A	0.7514	1.1065	0.1979	0.047*	
H4B	0.8872	1.2394	0.2675	0.047*	
C5	0.7574 (3)	1.1697 (3)	0.3619 (3)	0.0396 (8)	
C6	0.7087 (4)	1.2867 (3)	0.3612 (3)	0.0550 (10)	
H6A	0.7895	1.3723	0.3820	0.083*	
H6B	0.6558	1.2915	0.4122	0.083*	
H6C	0.6506	1.2692	0.2892	0.083*	
C7	0.8677 (4)	1.2122 (4)	0.4726 (3)	0.0531 (9)	
H7A	0.9000	1.1409	0.4751	0.080*	
H7B	0.8264	1.2266	0.5304	0.080*	
H7C	0.9459	1.2957	0.4817	0.080*	
C8	0.6332 (3)	1.0379 (3)	0.3445 (3)	0.0386 (8)	
C9	0.6119 (4)	0.9614 (3)	0.4176 (3)	0.0443 (8)	
H9	0.6769	0.9818	0.4845	0.053*	
C10	0.4812 (4)	0.8516 (4)	0.3765 (3)	0.0474 (9)	
H10	0.4471	0.7835	0.4080	0.057*	
C11	0.4082 (4)	0.8617 (3)	0.2776 (3)	0.0454 (8)	
C12	0.5011 (4)	0.9761 (3)	0.2561 (3)	0.0398 (8)	
C13	0.4532 (4)	1.0101 (3)	0.1619 (3)	0.0476 (9)	
H13	0.5130	1.0835	0.1458	0.057*	
C14	0.3188 (4)	0.9340 (4)	0.0952 (3)	0.0600 (10)	
H14	0.2864	0.9571	0.0343	0.072*	
C15	0.2277 (4)	0.8205 (4)	0.1171 (3)	0.0663 (11)	
H15	0.1364	0.7703	0.0701	0.080*	
C16	0.2693 (4)	0.7831 (4)	0.2038 (3)	0.0576 (10)	
H16	0.2083	0.7066	0.2158	0.069*	
C17	1.1715 (4)	1.4208 (4)	0.3664 (3)	0.0639 (11)	
H17A	1.0850	1.4302	0.3421	0.096*	
H17B	1.2488	1.4992	0.3660	0.096*	
H17C	1.1844	1.4149	0.4393	0.096*	
C18	1.3399 (4)	1.2627 (4)	0.3076 (4)	0.0705 (12)	
H18A	1.3647	1.2576	0.3804	0.106*	
H18B	1.4075	1.3453	0.3034	0.106*	
H18C	1.3392	1.1845	0.2566	0.106*	
C19	1.0933 (5)	1.2434 (5)	0.1285 (3)	0.0937 (17)	
H19A	1.1033	1.1674	0.0837	0.141*	
H19B	1.1440	1.3258	0.1136	0.141*	
H19C	0.9954	1.2265	0.1124	0.141*	
C20	0.6892 (6)	0.5070 (4)	0.1915 (4)	0.0815 (15)	
H20A	0.7063	0.5130	0.2682	0.098*	
H20B	0.5925	0.4431	0.1522	0.098*	
C21	0.7848 (6)	0.4608 (5)	0.1475 (5)	0.0927 (17)	
H21A	0.7346	0.3666	0.0995	0.111*	
H21B	0.8587	0.4645	0.2064	0.111*	
C22	0.8460 (5)	0.5506 (4)	0.0862 (4)	0.0704 (12)	
H22A	0.8309	0.4973	0.0120	0.085*	
H22B	0.9464	0.6030	0.1213	0.085*	
C23	0.7710 (4)	0.6442 (4)	0.0868 (3)	0.0541 (10)	
H23A	0.8359	0.7368	0.0961	0.065*	
H23B	0.6958	0.6117	0.0188	0.065*	

### Atomic displacement parameters (Å^2^)

**Table:**

	*U*^11^	*U*^22^	*U*^33^	*U*^12^	*U*^13^	*U*^23^
Zr1	0.03764 (19)	0.03099 (18)	0.03008 (17)	0.01108 (14)	0.01052 (13)	0.01184 (13)
Cl1	0.0690 (6)	0.0556 (5)	0.0356 (4)	0.0332 (5)	0.0162 (4)	0.0191 (4)
Cl2	0.0513 (6)	0.0393 (5)	0.0695 (6)	0.0047 (4)	0.0176 (5)	0.0127 (5)
Cl3	0.0765 (7)	0.0664 (6)	0.0411 (5)	0.0357 (5)	0.0168 (5)	0.0286 (5)
Si1	0.0380 (5)	0.0431 (5)	0.0452 (5)	0.0107 (4)	0.0134 (4)	0.0151 (4)
O1	0.0578 (15)	0.0310 (12)	0.0447 (13)	0.0179 (11)	0.0201 (11)	0.0155 (10)
N1	0.0359 (15)	0.0346 (14)	0.0433 (15)	0.0148 (12)	0.0134 (12)	0.0144 (12)
N2	0.0402 (16)	0.0342 (15)	0.0379 (15)	0.0158 (13)	0.0088 (12)	0.0130 (12)
C1	0.0377 (19)	0.0373 (18)	0.0351 (17)	0.0167 (15)	0.0120 (15)	0.0133 (14)
C2	0.0365 (19)	0.042 (2)	0.056 (2)	0.0199 (17)	0.0105 (16)	0.0130 (17)
C3	0.046 (2)	0.0373 (19)	0.049 (2)	0.0212 (17)	0.0092 (17)	0.0112 (16)
C4	0.0344 (18)	0.0351 (18)	0.0488 (19)	0.0127 (15)	0.0122 (15)	0.0164 (15)
C5	0.0403 (19)	0.0336 (17)	0.0412 (18)	0.0164 (15)	0.0087 (15)	0.0061 (15)
C6	0.053 (2)	0.043 (2)	0.072 (3)	0.0251 (18)	0.020 (2)	0.0125 (19)
C7	0.048 (2)	0.048 (2)	0.045 (2)	0.0122 (18)	0.0049 (17)	0.0010 (17)
C8	0.0371 (19)	0.0379 (18)	0.0396 (18)	0.0159 (15)	0.0128 (15)	0.0080 (15)
C9	0.051 (2)	0.047 (2)	0.0324 (17)	0.0199 (18)	0.0137 (16)	0.0091 (15)
C10	0.051 (2)	0.048 (2)	0.049 (2)	0.0180 (18)	0.0258 (18)	0.0198 (17)
C11	0.041 (2)	0.045 (2)	0.053 (2)	0.0183 (17)	0.0202 (17)	0.0128 (17)
C12	0.0380 (19)	0.0416 (19)	0.0428 (18)	0.0204 (16)	0.0141 (15)	0.0107 (15)
C13	0.048 (2)	0.043 (2)	0.052 (2)	0.0216 (18)	0.0114 (18)	0.0143 (17)
C14	0.056 (3)	0.063 (3)	0.057 (2)	0.031 (2)	0.001 (2)	0.016 (2)
C15	0.043 (2)	0.067 (3)	0.072 (3)	0.019 (2)	0.001 (2)	0.013 (2)
C16	0.039 (2)	0.055 (2)	0.072 (3)	0.0133 (19)	0.015 (2)	0.017 (2)
C17	0.061 (3)	0.040 (2)	0.086 (3)	0.0140 (19)	0.024 (2)	0.019 (2)
C18	0.049 (2)	0.066 (3)	0.099 (3)	0.020 (2)	0.035 (2)	0.028 (3)
C19	0.084 (4)	0.101 (4)	0.060 (3)	−0.005 (3)	0.013 (2)	0.039 (3)
C20	0.118 (4)	0.047 (2)	0.117 (4)	0.047 (3)	0.069 (3)	0.045 (3)
C21	0.116 (4)	0.080 (3)	0.138 (5)	0.069 (3)	0.076 (4)	0.057 (3)
C22	0.080 (3)	0.081 (3)	0.078 (3)	0.052 (3)	0.035 (3)	0.035 (3)
C23	0.073 (3)	0.053 (2)	0.043 (2)	0.029 (2)	0.0262 (19)	0.0146 (17)

### Geometric parameters (Å, °)

**Table:**

Zr1—N2	2.338 (3)	C8—C9	1.408 (4)
Zr1—O1	2.375 (2)	C8—C12	1.457 (4)
Zr1—Cl1	2.4647 (8)	C9—C10	1.384 (5)
Zr1—C10	2.472 (3)	C9—H9	0.9300
Zr1—Cl2	2.4720 (9)	C10—C11	1.424 (5)
Zr1—Cl3	2.4898 (9)	C10—H10	0.9300
Zr1—C9	2.509 (3)	C11—C12	1.424 (5)
Zr1—C8	2.622 (3)	C11—C16	1.427 (5)
Zr1—C11	2.642 (3)	C12—C13	1.420 (5)
Zr1—C12	2.720 (3)	C13—C14	1.361 (5)
Si1—N1	1.814 (3)	C13—H13	0.9300
Si1—C18	1.833 (4)	C14—C15	1.411 (6)
Si1—C19	1.842 (4)	C14—H14	0.9300
Si1—C17	1.843 (4)	C15—C16	1.339 (5)
O1—C20	1.453 (4)	C15—H15	0.9300
O1—C23	1.462 (4)	C16—H16	0.9300
N1—C1	1.356 (4)	C17—H17A	0.9600
N1—C2	1.392 (4)	C17—H17B	0.9600
N2—C1	1.329 (4)	C17—H17C	0.9600
N2—C3	1.390 (4)	C18—H18A	0.9600
C1—C4	1.488 (4)	C18—H18B	0.9600
C2—C3	1.335 (5)	C18—H18C	0.9600
C2—H2	0.9300	C19—H19A	0.9600
C3—H3	0.9300	C19—H19B	0.9600
C4—C5	1.563 (4)	C19—H19C	0.9600
C4—H4A	0.9700	C20—C21	1.445 (6)
C4—H4B	0.9700	C20—H20A	0.9700
C5—C8	1.511 (4)	C20—H20B	0.9700
C5—C7	1.534 (5)	C21—C22	1.465 (6)
C5—C6	1.541 (4)	C21—H21A	0.9700
C6—H6A	0.9600	C21—H21B	0.9700
C6—H6B	0.9600	C22—C23	1.503 (5)
C6—H6C	0.9600	C22—H22A	0.9700
C7—H7A	0.9600	C22—H22B	0.9700
C7—H7B	0.9600	C23—H23A	0.9700
C7—H7C	0.9600	C23—H23B	0.9700
			
N2—Zr1—O1	77.04 (8)	H7A—C7—H7B	109.5
N2—Zr1—Cl1	84.08 (6)	C5—C7—H7C	109.5
O1—Zr1—Cl1	79.04 (6)	H7A—C7—H7C	109.5
N2—Zr1—C10	120.70 (10)	H7B—C7—H7C	109.5
O1—Zr1—C10	151.10 (10)	C9—C8—C12	105.6 (3)
Cl1—Zr1—C10	122.63 (9)	C9—C8—C5	127.4 (3)
N2—Zr1—Cl2	155.51 (7)	C12—C8—C5	126.4 (3)
O1—Zr1—Cl2	78.56 (6)	C9—C8—Zr1	69.73 (17)
Cl1—Zr1—Cl2	93.30 (3)	C12—C8—Zr1	77.94 (18)
C10—Zr1—Cl2	81.10 (8)	C5—C8—Zr1	124.3 (2)
N2—Zr1—Cl3	81.90 (7)	C10—C9—C8	110.9 (3)
O1—Zr1—Cl3	76.92 (6)	C10—C9—Zr1	72.37 (18)
Cl1—Zr1—Cl3	154.33 (3)	C8—C9—Zr1	78.51 (18)
C10—Zr1—Cl3	83.04 (9)	C10—C9—H9	124.5
Cl2—Zr1—Cl3	90.66 (3)	C8—C9—H9	124.5
N2—Zr1—C9	88.44 (10)	Zr1—C9—H9	116.3
O1—Zr1—C9	151.65 (10)	C9—C10—C11	107.8 (3)
Cl1—Zr1—C9	124.05 (8)	C9—C10—Zr1	75.37 (19)
C10—Zr1—C9	32.26 (11)	C11—C10—Zr1	80.5 (2)
Cl2—Zr1—C9	112.69 (8)	C9—C10—H10	126.1
Cl3—Zr1—C9	77.01 (8)	C11—C10—H10	126.1
N2—Zr1—C8	75.20 (9)	Zr1—C10—H10	110.7
O1—Zr1—C8	151.85 (9)	C10—C11—C12	107.7 (3)
Cl1—Zr1—C8	93.50 (7)	C10—C11—C16	132.9 (3)
C10—Zr1—C8	53.60 (11)	C12—C11—C16	119.4 (3)
Cl2—Zr1—C8	129.29 (8)	C10—C11—Zr1	67.36 (19)
Cl3—Zr1—C8	103.50 (7)	C12—C11—Zr1	77.67 (19)
C9—Zr1—C8	31.77 (10)	C16—C11—Zr1	121.4 (2)
N2—Zr1—C11	127.00 (10)	C13—C12—C11	119.2 (3)
O1—Zr1—C11	153.12 (10)	C13—C12—C8	133.1 (3)
Cl1—Zr1—C11	90.77 (8)	C11—C12—C8	107.7 (3)
C10—Zr1—C11	32.11 (11)	C13—C12—Zr1	124.3 (2)
Cl2—Zr1—C11	77.27 (8)	C11—C12—Zr1	71.58 (19)
Cl3—Zr1—C11	114.83 (8)	C8—C12—Zr1	70.47 (18)
C9—Zr1—C11	52.20 (11)	C14—C13—C12	119.3 (3)
C8—Zr1—C11	52.45 (10)	C14—C13—H13	120.4
N2—Zr1—C12	98.51 (9)	C12—C13—H13	120.4
O1—Zr1—C12	153.90 (9)	C13—C14—C15	121.1 (4)
Cl1—Zr1—C12	74.91 (7)	C13—C14—H14	119.5
C10—Zr1—C12	52.31 (11)	C15—C14—H14	119.5
Cl2—Zr1—C12	104.31 (8)	C16—C15—C14	121.7 (4)
Cl3—Zr1—C12	128.42 (7)	C16—C15—H15	119.2
C9—Zr1—C12	51.57 (10)	C14—C15—H15	119.2
C8—Zr1—C12	31.59 (9)	C15—C16—C11	119.4 (4)
C11—Zr1—C12	30.75 (10)	C15—C16—H16	120.3
N1—Si1—C18	106.39 (16)	C11—C16—H16	120.3
N1—Si1—C19	104.76 (17)	Si1—C17—H17A	109.5
C18—Si1—C19	113.1 (2)	Si1—C17—H17B	109.5
N1—Si1—C17	111.00 (15)	H17A—C17—H17B	109.5
C18—Si1—C17	108.7 (2)	Si1—C17—H17C	109.5
C19—Si1—C17	112.6 (2)	H17A—C17—H17C	109.5
C20—O1—C23	105.4 (3)	H17B—C17—H17C	109.5
C20—O1—Zr1	125.3 (2)	Si1—C18—H18A	109.5
C23—O1—Zr1	126.99 (18)	Si1—C18—H18B	109.5
C1—N1—C2	105.3 (3)	H18A—C18—H18B	109.5
C1—N1—Si1	129.6 (2)	Si1—C18—H18C	109.5
C2—N1—Si1	124.4 (2)	H18A—C18—H18C	109.5
C1—N2—C3	104.8 (3)	H18B—C18—H18C	109.5
C1—N2—Zr1	130.6 (2)	Si1—C19—H19A	109.5
C3—N2—Zr1	124.4 (2)	Si1—C19—H19B	109.5
N2—C1—N1	112.3 (3)	H19A—C19—H19B	109.5
N2—C1—C4	123.7 (3)	Si1—C19—H19C	109.5
N1—C1—C4	123.7 (3)	H19A—C19—H19C	109.5
C3—C2—N1	107.6 (3)	H19B—C19—H19C	109.5
C3—C2—H2	126.2	C21—C20—O1	107.4 (3)
N1—C2—H2	126.2	C21—C20—H20A	110.2
C2—C3—N2	109.9 (3)	O1—C20—H20A	110.2
C2—C3—H3	125.0	C21—C20—H20B	110.2
N2—C3—H3	125.0	O1—C20—H20B	110.2
C1—C4—C5	111.8 (3)	H20A—C20—H20B	108.5
C1—C4—H4A	109.3	C20—C21—C22	108.4 (4)
C5—C4—H4A	109.3	C20—C21—H21A	110.0
C1—C4—H4B	109.3	C22—C21—H21A	110.0
C5—C4—H4B	109.3	C20—C21—H21B	110.0
H4A—C4—H4B	107.9	C22—C21—H21B	110.0
C8—C5—C7	110.9 (3)	H21A—C21—H21B	108.4
C8—C5—C6	110.0 (3)	C21—C22—C23	105.2 (3)
C7—C5—C6	107.8 (3)	C21—C22—H22A	110.7
C8—C5—C4	109.5 (2)	C23—C22—H22A	110.7
C7—C5—C4	109.8 (3)	C21—C22—H22B	110.7
C6—C5—C4	108.7 (3)	C23—C22—H22B	110.7
C5—C6—H6A	109.5	H22A—C22—H22B	108.8
C5—C6—H6B	109.5	O1—C23—C22	105.5 (3)
H6A—C6—H6B	109.5	O1—C23—H23A	110.6
C5—C6—H6C	109.5	C22—C23—H23A	110.6
H6A—C6—H6C	109.5	O1—C23—H23B	110.6
H6B—C6—H6C	109.5	C22—C23—H23B	110.6
C5—C7—H7A	109.5	H23A—C23—H23B	108.8
C5—C7—H7B	109.5		
			
N2—Zr1—O1—C20	137.0 (3)	Cl2—Zr1—C9—C8	−128.95 (18)
Cl1—Zr1—O1—C20	−136.6 (3)	Cl3—Zr1—C9—C8	145.9 (2)
C10—Zr1—O1—C20	5.1 (4)	C11—Zr1—C9—C8	−77.7 (2)
Cl2—Zr1—O1—C20	−41.0 (3)	C12—Zr1—C9—C8	−38.36 (19)
Cl3—Zr1—O1—C20	52.4 (3)	C8—C9—C10—C11	−5.0 (4)
C9—Zr1—O1—C20	76.0 (4)	Zr1—C9—C10—C11	−74.8 (2)
C8—Zr1—O1—C20	146.7 (3)	C8—C9—C10—Zr1	69.8 (2)
C11—Zr1—O1—C20	−67.2 (4)	N2—Zr1—C10—C9	0.4 (2)
C12—Zr1—O1—C20	−140.2 (3)	O1—Zr1—C10—C9	122.8 (2)
N2—Zr1—O1—C23	−62.6 (3)	Cl1—Zr1—C10—C9	−103.4 (2)
Cl1—Zr1—O1—C23	23.7 (3)	Cl2—Zr1—C10—C9	168.4 (2)
C10—Zr1—O1—C23	165.5 (3)	Cl3—Zr1—C10—C9	76.6 (2)
Cl2—Zr1—O1—C23	119.4 (3)	C8—Zr1—C10—C9	−35.82 (19)
Cl3—Zr1—O1—C23	−147.2 (3)	C11—Zr1—C10—C9	−111.4 (3)
C9—Zr1—O1—C23	−123.6 (3)	C12—Zr1—C10—C9	−75.7 (2)
C8—Zr1—O1—C23	−53.0 (4)	N2—Zr1—C10—C11	111.8 (2)
C11—Zr1—O1—C23	93.1 (3)	O1—Zr1—C10—C11	−125.8 (2)
C12—Zr1—O1—C23	20.2 (4)	Cl1—Zr1—C10—C11	8.0 (2)
C18—Si1—N1—C1	−179.5 (3)	Cl2—Zr1—C10—C11	−80.22 (19)
C19—Si1—N1—C1	−59.5 (3)	Cl3—Zr1—C10—C11	−172.0 (2)
C17—Si1—N1—C1	62.4 (3)	C9—Zr1—C10—C11	111.4 (3)
C18—Si1—N1—C2	−10.9 (3)	C8—Zr1—C10—C11	75.5 (2)
C19—Si1—N1—C2	109.2 (3)	C12—Zr1—C10—C11	35.71 (19)
C17—Si1—N1—C2	−129.0 (3)	C9—C10—C11—C12	3.3 (4)
O1—Zr1—N2—C1	139.2 (3)	Zr1—C10—C11—C12	−67.9 (2)
Cl1—Zr1—N2—C1	59.1 (3)	C9—C10—C11—C16	−176.3 (4)
C10—Zr1—N2—C1	−65.6 (3)	Zr1—C10—C11—C16	112.6 (4)
Cl2—Zr1—N2—C1	144.0 (2)	C9—C10—C11—Zr1	71.2 (2)
Cl3—Zr1—N2—C1	−142.5 (3)	N2—Zr1—C11—C10	−90.1 (2)
C9—Zr1—N2—C1	−65.4 (3)	O1—Zr1—C11—C10	119.9 (2)
C8—Zr1—N2—C1	−36.1 (3)	Cl1—Zr1—C11—C10	−173.27 (19)
C11—Zr1—N2—C1	−27.4 (3)	Cl2—Zr1—C11—C10	93.5 (2)
C12—Zr1—N2—C1	−14.6 (3)	Cl3—Zr1—C11—C10	8.8 (2)
O1—Zr1—N2—C3	−34.1 (2)	C9—Zr1—C11—C10	−38.98 (19)
Cl1—Zr1—N2—C3	−114.2 (2)	C8—Zr1—C11—C10	−79.4 (2)
C10—Zr1—N2—C3	121.2 (2)	C12—Zr1—C11—C10	−115.4 (3)
Cl2—Zr1—N2—C3	−29.2 (3)	N2—Zr1—C11—C12	25.3 (2)
Cl3—Zr1—N2—C3	44.3 (2)	O1—Zr1—C11—C12	−124.7 (2)
C9—Zr1—N2—C3	121.4 (2)	Cl1—Zr1—C11—C12	−57.87 (19)
C8—Zr1—N2—C3	150.6 (3)	C10—Zr1—C11—C12	115.4 (3)
C11—Zr1—N2—C3	159.3 (2)	Cl2—Zr1—C11—C12	−151.1 (2)
C12—Zr1—N2—C3	172.1 (2)	Cl3—Zr1—C11—C12	124.17 (18)
C3—N2—C1—N1	0.1 (3)	C9—Zr1—C11—C12	76.4 (2)
Zr1—N2—C1—N1	−174.11 (19)	C8—Zr1—C11—C12	35.96 (18)
C3—N2—C1—C4	−174.0 (3)	N2—Zr1—C11—C16	142.3 (3)
Zr1—N2—C1—C4	11.8 (4)	O1—Zr1—C11—C16	−7.6 (4)
C2—N1—C1—N2	0.7 (3)	Cl1—Zr1—C11—C16	59.2 (3)
Si1—N1—C1—N2	171.1 (2)	C10—Zr1—C11—C16	−127.5 (4)
C2—N1—C1—C4	174.8 (3)	Cl2—Zr1—C11—C16	−34.0 (3)
Si1—N1—C1—C4	−14.8 (4)	Cl3—Zr1—C11—C16	−118.8 (3)
C1—N1—C2—C3	−1.3 (4)	C9—Zr1—C11—C16	−166.5 (4)
Si1—N1—C2—C3	−172.3 (2)	C8—Zr1—C11—C16	153.0 (3)
N1—C2—C3—N2	1.5 (4)	C12—Zr1—C11—C16	117.1 (4)
C1—N2—C3—C2	−1.0 (4)	C10—C11—C12—C13	−179.3 (3)
Zr1—N2—C3—C2	173.7 (2)	C16—C11—C12—C13	0.3 (5)
N2—C1—C4—C5	55.6 (4)	Zr1—C11—C12—C13	119.6 (3)
N1—C1—C4—C5	−117.8 (3)	C10—C11—C12—C8	−0.5 (4)
C1—C4—C5—C8	−74.1 (3)	C16—C11—C12—C8	179.1 (3)
C1—C4—C5—C7	47.9 (4)	Zr1—C11—C12—C8	−61.6 (2)
C1—C4—C5—C6	165.6 (3)	C10—C11—C12—Zr1	61.1 (2)
C7—C5—C8—C9	2.0 (4)	C16—C11—C12—Zr1	−119.3 (3)
C6—C5—C8—C9	−117.1 (4)	C9—C8—C12—C13	176.2 (3)
C4—C5—C8—C9	123.5 (3)	C5—C8—C12—C13	4.8 (6)
C7—C5—C8—C12	171.5 (3)	Zr1—C8—C12—C13	−119.1 (4)
C6—C5—C8—C12	52.4 (4)	C9—C8—C12—C11	−2.4 (4)
C4—C5—C8—C12	−67.1 (4)	C5—C8—C12—C11	−173.7 (3)
C7—C5—C8—Zr1	−87.6 (3)	Zr1—C8—C12—C11	62.3 (2)
C6—C5—C8—Zr1	153.3 (2)	C9—C8—C12—Zr1	−64.7 (2)
C4—C5—C8—Zr1	33.8 (3)	C5—C8—C12—Zr1	124.0 (3)
N2—Zr1—C8—C9	−111.9 (2)	N2—Zr1—C12—C13	86.9 (3)
O1—Zr1—C8—C9	−121.7 (2)	O1—Zr1—C12—C13	9.0 (4)
Cl1—Zr1—C8—C9	165.2 (2)	Cl1—Zr1—C12—C13	5.4 (3)
C10—Zr1—C8—C9	36.4 (2)	C10—Zr1—C12—C13	−150.7 (3)
Cl2—Zr1—C8—C9	68.0 (2)	Cl2—Zr1—C12—C13	−84.2 (3)
Cl3—Zr1—C8—C9	−34.2 (2)	Cl3—Zr1—C12—C13	173.3 (2)
C11—Zr1—C8—C9	76.9 (2)	C9—Zr1—C12—C13	168.0 (3)
C12—Zr1—C8—C9	111.9 (3)	C8—Zr1—C12—C13	129.4 (4)
N2—Zr1—C8—C12	136.2 (2)	C11—Zr1—C12—C13	−113.3 (4)
O1—Zr1—C8—C12	126.5 (2)	N2—Zr1—C12—C11	−159.8 (2)
Cl1—Zr1—C8—C12	53.30 (18)	O1—Zr1—C12—C11	122.3 (2)
C10—Zr1—C8—C12	−75.5 (2)	Cl1—Zr1—C12—C11	118.7 (2)
Cl2—Zr1—C8—C12	−43.9 (2)	C10—Zr1—C12—C11	−37.4 (2)
Cl3—Zr1—C8—C12	−146.08 (17)	Cl2—Zr1—C12—C11	29.1 (2)
C9—Zr1—C8—C12	−111.9 (3)	Cl3—Zr1—C12—C11	−73.4 (2)
C11—Zr1—C8—C12	−34.97 (18)	C9—Zr1—C12—C11	−78.7 (2)
N2—Zr1—C8—C5	10.2 (2)	C8—Zr1—C12—C11	−117.3 (3)
O1—Zr1—C8—C5	0.4 (4)	N2—Zr1—C12—C8	−42.55 (19)
Cl1—Zr1—C8—C5	−72.7 (2)	O1—Zr1—C12—C8	−120.4 (2)
C10—Zr1—C8—C5	158.5 (3)	Cl1—Zr1—C12—C8	−124.02 (19)
Cl2—Zr1—C8—C5	−169.9 (2)	C10—Zr1—C12—C8	79.9 (2)
Cl3—Zr1—C8—C5	87.9 (2)	Cl2—Zr1—C12—C8	146.39 (17)
C9—Zr1—C8—C5	122.1 (4)	Cl3—Zr1—C12—C8	43.8 (2)
C11—Zr1—C8—C5	−161.0 (3)	C9—Zr1—C12—C8	38.59 (18)
C12—Zr1—C8—C5	−126.0 (3)	C11—Zr1—C12—C8	117.3 (3)
C12—C8—C9—C10	4.6 (4)	C11—C12—C13—C14	1.2 (5)
C5—C8—C9—C10	175.8 (3)	C8—C12—C13—C14	−177.3 (4)
Zr1—C8—C9—C10	−65.9 (2)	Zr1—C12—C13—C14	87.9 (4)
C12—C8—C9—Zr1	70.5 (2)	C12—C13—C14—C15	−1.4 (6)
C5—C8—C9—Zr1	−118.3 (3)	C13—C14—C15—C16	0.0 (6)
N2—Zr1—C9—C10	−179.7 (2)	C14—C15—C16—C11	1.5 (6)
O1—Zr1—C9—C10	−121.2 (2)	C10—C11—C16—C15	177.9 (4)
Cl1—Zr1—C9—C10	98.5 (2)	C12—C11—C16—C15	−1.6 (6)
Cl2—Zr1—C9—C10	−12.4 (2)	Zr1—C11—C16—C15	−94.9 (4)
Cl3—Zr1—C9—C10	−97.6 (2)	C23—O1—C20—C21	25.8 (5)
C8—Zr1—C9—C10	116.5 (3)	Zr1—O1—C20—C21	−170.3 (3)
C11—Zr1—C9—C10	38.8 (2)	O1—C20—C21—C22	−12.5 (6)
C12—Zr1—C9—C10	78.2 (2)	C20—C21—C22—C23	−5.4 (6)
N2—Zr1—C9—C8	63.8 (2)	C20—O1—C23—C22	−28.9 (4)
O1—Zr1—C9—C8	122.3 (2)	Zr1—O1—C23—C22	167.7 (2)
Cl1—Zr1—C9—C8	−18.0 (2)	C21—C22—C23—O1	21.1 (5)
C10—Zr1—C9—C8	−116.5 (3)		
